# Immunomodulatory Strategies for Managing Viral Infections in Solid Organ Transplantation: Progress and Challenges

**DOI:** 10.1007/s00284-026-04898-y

**Published:** 2026-04-20

**Authors:** Amir Elalouf, Hanan Maoz

**Affiliations:** Department of Management, Bar-Ilan University, 5290002 Ramat Gan, Israel

## Abstract

Solid organ transplantation (SOT) is a critical treatment for end-stage organ failure. Still, lifelong immunosuppression leaves recipients vulnerable to opportunistic viral infections, which can lead to severe complications such as graft dysfunction and post-transplant lymphoproliferative disorder. With emerging viral threats such as SARS-CoV-2 and arboviruses, alongside persistent challenges posed by CMV, EBV, and BKV, this review is timely in addressing the evolving landscape of post-transplant viral infections and their management. Recent studies highlight how immunosuppression impairs both innate and adaptive antiviral defenses, including diminished toll-like receptor signaling, dysfunction of NK cells, and disrupted T- and B-cell responses. Viruses exploit these deficits through immune evasion strategies, such as MHC-I downregulation and the production of immunosuppressive microRNA. Advances in management include antiviral prophylaxis, adoptive T-cell therapy, and immune monitoring, with emerging therapies like virus-specific T-cell infusions and complement inhibition showing promise. The findings underscore the need for personalized, organ-specific approaches to post-transplant care. Enhanced surveillance, vaccination strategies, and novel immunotherapies are critical in mitigating viral risks. Future research should focus on immune-risk stratification and the development of an adaptable therapeutic approach to enhance transplant outcomes in the face of evolving viral threats.

## Introduction

Solid organ transplantation (SOT), a transformative medical intervention, is often a critical solution for patients facing organ failure, offering them a chance to regain health and improve their quality of life [102]. Commonly transplanted organs include the kidney, liver, heart, lung, pancreas, and intestine, with advanced procedures like face and hand transplants also being performed in specialized cases. Although SOT poses a significant challenge, the recipient’s immune system may identify the transplant as foreign and mount an immune response to reject it [40]. To counteract this, patients must take immunosuppressive medications to dampen their immune system’s activity, reducing the risk of rejection [110]. Choosing to undergo a transplant is a complex decision shaped by a person’s age, health, and history, and it marks the beginning of lifelong care, with medications, treatments, and checkups to keep the new organ working well [26, 49].

Managing viral infections in SOT recipients requires a balanced approach that considers immune suppression, virus-specific T cell responses, and both prevention and treatment. Prolonged immunosuppression weakens the immune system, increasing vulnerability to new infections and viral reactivations [97]. Cytotoxic T lymphocytes (CD8+ T cells), crucial for clearing infected cells, often recover slowly after transplantation, increasing infection risk [115]. Adaptive immunity, including antibody production, is also affected, sometimes altered by donor-derived antibodies or compatibility issues [129].

To address these risks, strategies such as vaccination and antiviral prophylaxis are critical. Early use of effective antiviral medications can help limit the extent of infection and prevent secondary complications [41]. An emerging area of interest is adoptive immunotherapy, which involves infusing virus-specific T cells derived from either healthy donors or the patient themselves. This method has shown encouraging results in controlling viral infections post-transplant. Additionally, advancements in immune monitoring now enable identification of patients at higher risk, allowing for more tailored, timely treatment plans [105].

This review aims to provide a comprehensive overview of the mechanisms through which viral infections impact SOT recipients, with a particular focus on the interplay between viral pathogenesis and the host immune response under immunosuppression. It discusses the most prevalent viral threats, including cytomegalovirus (CMV), Epstein-Barr virus (EBV), BK polyomavirus (BKV), hepatitis B and C (HBV/HCV), and herpes viruses such as HSV and VZV, as well as emerging pathogens such as SARS-CoV-2. It explores how these infections can lead to graft dysfunction or rejection.

## Recent Trends in Organ Transplantation

As of early 2025, organ transplantation continues to serve as a critical therapeutic modality for patients with end-stage organ failure, with recent data reflecting both advancements in transplantation practices and persistent disparities in organ supply and demand (Table 1). In 2023, around 172,000 organ transplants were performed globally, with kidney transplants making up nearly 65%, highlighting the continued dominance of renal replacement therapy [36]. While full 2024 global data are still emerging, early reports suggest a steady rise in transplant activity. In the U.S., 104,753 people were on the waiting list by December 2024, with kidneys being the most needed [56]. The U.S. performed over 48,000 transplants in 2024, a 3.3% increase from 2023, including a record number of transplants from deceased donors [154]. The Eurotransplant region also saw high activity, with over 7,100 transplants from deceased donors in 2024 and 3,364 more in early 2025 [37].

**Table 1:** Organ transplantation activity, donor availability, and waiting list burden in selected countries and regions (2024)

Country/region	Wait-list (2024)	Donors available (2024)	Transplants performed (2024)	Reference
United States	~104,753	23,373 deceased, 7,030 living	48,149	[7, 56, 118]
Spain	~5,000–6,000	2,562 deceased, 404 living	6,464	[93, 123]
Eurotransplant Region (8 countries)	13,570	2,181 deceased (utilized)	7,150	[37]
Canada	4,044	Not specified (est. ~900–1,000)	3,454	[23, 24]
UK	~7,000 (varies by organ)	~1,500 deceased (utilized)	4,813	[38, 155]
Germany	~8,500	965 deceased (utilized)	3,646	[38, 155]
Italy	~8,000	1,731 deceased (utilized)	4,543	[38, 155]
France	~21,000	1,791 deceased (utilized)	5,450	[38, 155]
Israel	~1,481 patients	107 deceased donors	656 total transplants (kidney, heart, etc.)	[10, 64]

## Solid Organ Transplantation and Immune Responses Under Immunosuppression

After SOT, the recipient’s immune system recognizes the graft as foreign through alloantigens and damage-associated molecular patterns (DAMPs) released during ischemia-reperfusion injury. This realization activates both innate (macrophages, dendritic cells, neutrophils) and adaptive (T and B lymphocytes) responses, with T cells playing a central role in acute and chronic rejection. Memory T cells can drive repeated attacks, leading to graft damage. Natural killer (NK) cells also contribute to rejection and, interestingly, can mediate graft rejection independently of adaptive immunity. In IL-15-treated Rag-/- mice, which lack functional T and B cells, NK cells were shown to reject allogeneic skin grafts, underscoring their potential role in innate-driven graft injury [16, 88]. At the same time, when NK-cell activity is impaired by immunosuppression or intrinsic dysfunction, the risk of opportunistic infections rises significantly. Conversely, donor-derived NK cells residing in the graft may promote tolerance by eliminating recipient-reactive T cells [18, 65, 66, 75, 113, 133, 138, 146]. Inflammation facilitates immune-cell trafficking and influences rejection pathways depending on the organ type, graft duration, and patient immunologic risk [35, 48, 137]. The complement system further amplifies early graft injury through activation during ischemia-reperfusion and in antibody-mediated rejection [116, 122].

**Fig. 1:**
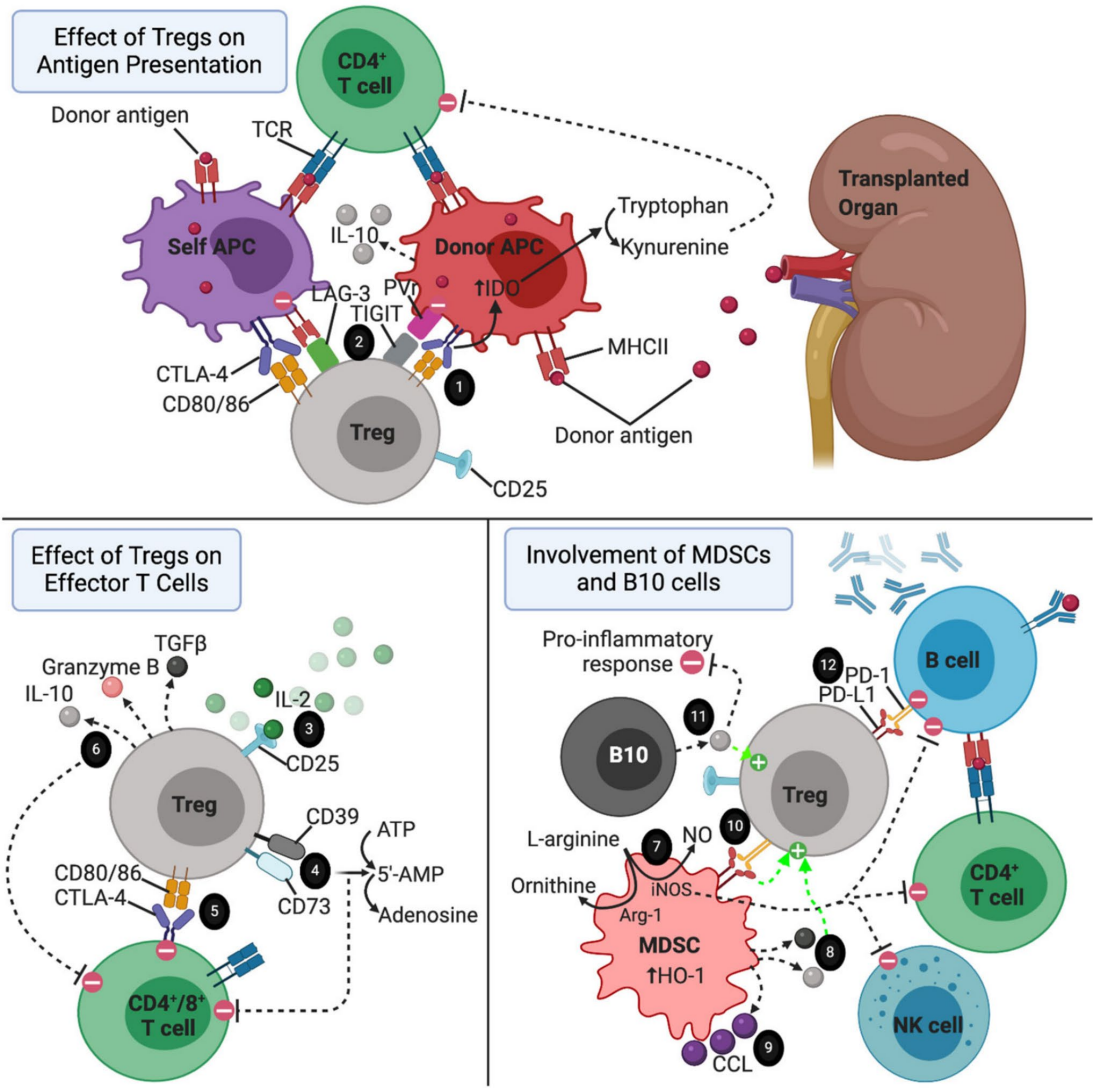
The immune landscape around a transplanted organ is shaped by a complex network of interactions aimed at maintaining tolerance and preventing rejection. Regulatory T cells (Tregs) play a central role in this environment. One of their key functions is to interact with antigen-presenting cells (APCs) through the CTLA-4 and CD80/86 pathways, which promote tryptophan catabolism by APCs via the IDO pathway, creating an immunosuppressive environment. Additionally, molecules such as LAG-3 and TIGIT, expressed on Tregs, help steer APCs toward a more tolerant, less inflammatory state. Tregs also help regulate immune responses by consuming IL-2, a cytokine essential for effector T cell activation, effectively limiting its availability. Enzymes such as CD39 and CD73, expressed on Tregs, convert extracellular ATP and 5’-AMP into adenosine, a molecule with anti-inflammatory properties. Beyond these molecular interactions, Tregs directly inhibit effector T cells by engaging the CTLA-4–CD80/86 axis and secreting anti-inflammatory cytokines, which dampen inflammation, promote cell death in overactive immune cells, and encourage the growth of other regulatory immune cells. Myeloid-derived suppressor cells (MDSCs) also contribute to this immune-regulating microenvironment. They suppress the activity of T cells, B cells, and NK cells by depleting L-arginine through an iNOS-dependent mechanism. This suppressive effect is further enhanced by the upregulation of enzymes such as arginase-1 (Arg-1) and heme oxygenase-1 (HO-1). MDSCs also support Treg activity by producing IL-10 and TGF-β—two cytokines known to encourage Treg expansion and function. Furthermore, MDSCs release the chemokine CCL5, which creates a directional gradient that draws Tregs to the graft site from peripheral tissues. The interaction between PD-L1 on MDSCs and PD-1 on Tregs strengthens Treg-mediated immune suppression. B10 regulatory B cells also contribute by releasing IL-10, which not only expands the Treg population but also broadly reduces inflammation. Finally, Tregs can induce programmed cell death in autoreactive B cells by engaging the PD-1 and PD-L1 signaling pathways, thereby maintaining immune homeostasis and protecting the graft from immune attack. Copyright © 2021 Oberholtzer, Atkinson, and Nadig [100], licensed under the terms of the Creative Commons Attribution License (CC BY)

The graft microenvironment relies on regulatory mechanisms for tolerance, including regulatory T cells (Tregs) that suppress responses via cell-contact, anti-inflammatory cytokines, and enzymes such as CD39/CD73. These are supported by myeloid-derived suppressor cells (MDSCs) and B10 cells, which produce IL-10 and recruit Tregs (Fig. 1) [100]. Trained immunity in innate cells (e.g., macrophages) can amplify responses to DAMPs or alloantigens, potentially intensifying rejection despite its protective role against pathogens [31, 101]. Current prevention strategies block immune activation at three signals: antigen recognition (signal 1), co-stimulation (signal 2), and cytokine release (signal 3) [75, 77, 114]. In multi-organ transplantation, interactions between grafts add further complexity, often requiring organ-specific approaches [69, 146].

Lifelong immunosuppression is essential to prevent rejection but impairs antiviral defenses, increasing susceptibility to opportunistic viruses (CMV, EBV, BKV). Modern regimens have evolved from azathioprine/corticosteroids to personalized combinations tailored by biomarkers and recipient factors [47, 111]. Tailoring prevention and treatment strategies to the specific transplant population, immune status, and associated risk factors is pivotal for effective management of viral infections post-transplantation. Such personalized approaches hold the potential to optimize outcomes and curtail infection rates [42]. Standard maintenance typically includes calcineurin inhibitors (CNI), antimetabolites, and corticosteroids. CNIs (cyclosporine A or tacrolimus) block calcineurin, preventing NFAT nuclear translocation and IL-2 production, thereby inhibiting T-cell proliferation and reducing graft rejection; the same mechanism limits virus-specific CD8⁺ T-cell expansion [59, 98, 131, 148]. Other agents include mTOR inhibitors (sirolimus, everolimus), purine analogs (mycophenolate), monoclonal antibodies (basiliximab), and glucocorticoids, each selected for efficacy, safety, and pharmacokinetics while balancing graft acceptance against infection risk. Vaccination responses and simultaneous multi-organ strategies further require individualized adjustments [126].

## Viral Infections in Solid Organ Transplantation

Viral infections are a major complication in solid organ transplantation (SOT) recipients. Post-transplant viral risk follows well-defined temporal phases: early (0–1 month, dominated by nosocomial and reactivation events), intermediate (1–6 months, peak period for opportunistic viruses such as CMV and EBV), and late (>6 months, driven by community-acquired and persistent infections) [34, 124]. Common pathogens include cytomegalovirus (CMV), Epstein-Barr virus (EBV), BK polyomavirus (BKV), hepatitis B and C viruses (HBV/HCV), herpes simplex virus (HSV), varicella-zoster virus (VZV), and emerging threats such as arboviruses and SARS-CoV-2 [34, 124]. Risk and severity vary by transplanted organ, surgical complexity, and environmental exposure (Table 2).

**Table 2:** Incidence of viral infections by organ type

Organ type	CMV incidence	EBV PTLD incidence	BKV incidence	HBV/HCV notes	HSV/VZV notes
Kidney	40%–100%	~2%	1%–10% BKVN	Low, managed with DAAs	Up to 70% without prophylaxis
Liver	Common	~2%	Rare	High risk, DAAs effective	Up to 70% without prophylaxis
Heart	9%–23%	4.9%–13%	Rare	Low, managed with DAAs	Up to 70% without prophylaxis
Lung	13% asymptomatic, 10% disease	High, varies	Rare	Low, managed with DAAs	Up to 70% without prophylaxis
Intestine	High risk	>10%	Rare	Low, managed with DAAs	Up to 70% without prophylaxis

Kidney recipients face high rates of CMV (40%–100%), with two-thirds progressing to disease in the first three months; the highest risk occurs in CMV-seronegative recipients of seropositive donor organs (D+/R−) (Table 3) [54]. CMV causes direct tissue-invasive disease and indirect effects, including allograft rejection and long-term graft dysfunction. Prophylaxis with valganciclovir or preemptive monitoring is standard; maribavir and letermovir are alternatives for cases resistant to valganciclovir [54, 57]. BKV is kidney-specific, causing BK virus-associated nephropathy (BKVN) in 1–10% of cases and may lead to graft loss; regular plasma/urine PCR screening guides immunosuppression adjustment (Table 3) [63]. EBV drives post-transplant lymphoproliferative disorder (PTLD) in ~2% of recipients overall, with higher rates in heart (~13%), lung, and intestinal transplants, particularly in EBV-seronegative recipients (Table 3) of seropositive organs [3, 132].

**Table 3:** High-prevalence viruses and key risks

Virus	Key risk/complication	Organ-specific notes
CMV	D+/R− highest risk	All organs are highest in lung/kidney
EBV	PTLD, varies by organ	Highest in lung/intestine, lowest in kidney
BKV	Nephropathy	Primarily kidney, 1%–10% incidence
HBV/HCV	Recurrence, managed with DAAs	Mainly liver, high historical recurrence
HSV/VZV	Mucocutaneous lesions, shingles	All organs, up to 70% without prophylaxis

In liver recipients, HBV recurrence is now rare with HBIG plus antivirals, while HCV is cured in >90% of cases with direct-acting antivirals (DAAs), enabling safe use of HCV-positive donors [71, 72]. HSV and VZV reactivation occurs in up to 70% without prophylaxis, manifesting as mucocutaneous lesions or shingles [153].

Heart, lung, and intestinal recipients show distinct patterns: heart transplants carry 9%–23% CMV risk and elevated PTLD; lung recipients experience frequent asymptomatic CMV (13%) plus respiratory viruses (influenza, RSV, parainfluenza) in up to 60% of cases in the first year; intestinal recipients have the highest PTLD rates (>10%) and overall viral burden (Table 3) [107].

Region-specific and emerging threats add complexity. In tropical areas, arboviruses (dengue, chikungunya, Zika) transmitted via natural exposure or the graft cause more severe disease, including acute kidney injury and neurological complications [94, 95]. The COVID-19 pandemic demonstrated markedly worse outcomes in SOT recipients, with 20%–30% mortality versus 0.8%–2% in the general population, even among vaccinated individuals, highlighting impaired vaccine responses [140].

## Innate And Adaptive Immune Responses Under Immunosuppression

Viral infections, particularly CMV, EBV, and BK virus, remain a major challenge in SOT recipients because immunosuppression and graft-related injury profoundly impair both innate and adaptive antiviral immunity (Table 4). These viruses are adept at exploiting weakened immune defenses.

**Table 4:** Key mechanisms of innate and adaptive immunity dysregulation in SOT

Mechanism	Description	Impact in SOT	Relevance to viral infections
TLR/RIG-I signaling impairment	TLRs activated by DAMPs, RIG-I plays a crucial role in antiviral responses, but there is potential impairment due to immunosuppression.	Inflammation, graft rejection, weakened antiviral defenses.	Increased susceptibility to viral reactivation.
NK cell cytotoxic dysfunction	NK cells contribute to rejection, are functionally impaired by immunosuppression, and have memory-like features affected.	Graft damage, increased infection risk.	Reduced control of virus-infected cells.
Complement hijacking	Viruses evade complement by mimicking regulators, not directly causing graft injury, but this is relevant for infections.	Persistent viral infections, potential graft complications.	Allows viruses to evade immune detection.
CD4⁺/CD8⁺ T-cell exhaustion	PD-1/CTLA-4 upregulation reduces T-cell function due to chronic antigen exposure.	Lower rejection risk, higher infection susceptibility.	Impaired CMV/EBV control, prolonged viremia.
B-cell maturation defects	Immunosuppression impairs B-cell differentiation, leading to hypogammaglobulinemia.	Increased infection risk, AMR via DSAs.	Reduced antibody response to CMV/EBV, PTLD risk.
Treg/Th17 imbalance	Th17 dominance promotes rejection; Tregs promote tolerance.	Higher rejection risk, potential tolerance induction.	Th17 inflammation worsens viral tissue damage.

### Innate Immunity Dysregulation

Innate immunity serves as the first line of defense against pathogens and is critical in the early post-transplant period for recognizing and responding to graft-related damage and infections. However, the transplantation process, including ischemia-reperfusion injury (IRI), surgical trauma, and the use of immunosuppressive drugs, can dysregulate these responses, leading to graft injury, rejection, and increased susceptibility to infections, particularly viral ones.

### TLR/RIG-I Signaling Impairment

Toll-like receptors (TLRs) are essential components of the innate immune system. As pattern recognition receptors (PRRs), they detect pathogen-associated molecular patterns (PAMPs) and DAMPs and initiate inflammatory signaling cascades in response. In SOT, DAMPs released as a result of ischemia-reperfusion injury (IRI) and surgical trauma can activate TLRs, particularly TLR4. This activation leads to the production of pro-inflammatory cytokines such as tumor necrosis factor-alpha (TNF-α), interleukin-1 (IL-1), and interleukin-6 (IL-6), creating a local immune environment that can promote graft rejection and interfere with immune tolerance mechanisms [147]. This impairment is particularly detrimental in the intermediate post-transplant period (1–6 months), when CMV and EBV reactivation peaks, because diminished TLR4/RIG-I signaling reduces type I interferon production and fails to adequately prime antiviral NK- and T-cell responses [130, 141].

Experimental studies support this mechanism. For example, mice with functional TLR4 signaling show significantly elevated levels of inflammatory cytokines following IRI, whereas TLR4-deficient mice exhibit reduced inflammation and improved graft outcomes, indicating a direct role of TLR4 in transplant-related injury [136]. Furthermore, TLR4 has been linked to endothelial dysfunction, considering an early event in the development of cardiac allograft vasculopathy (CAV). Clinical data from cardiac transplant recipients also support this, with 13 out of 38 patients showing increased TLR4 expression alongside elevated IL-12 and TNF-α during episodes of graft rejection [112].

**Fig. 2:**
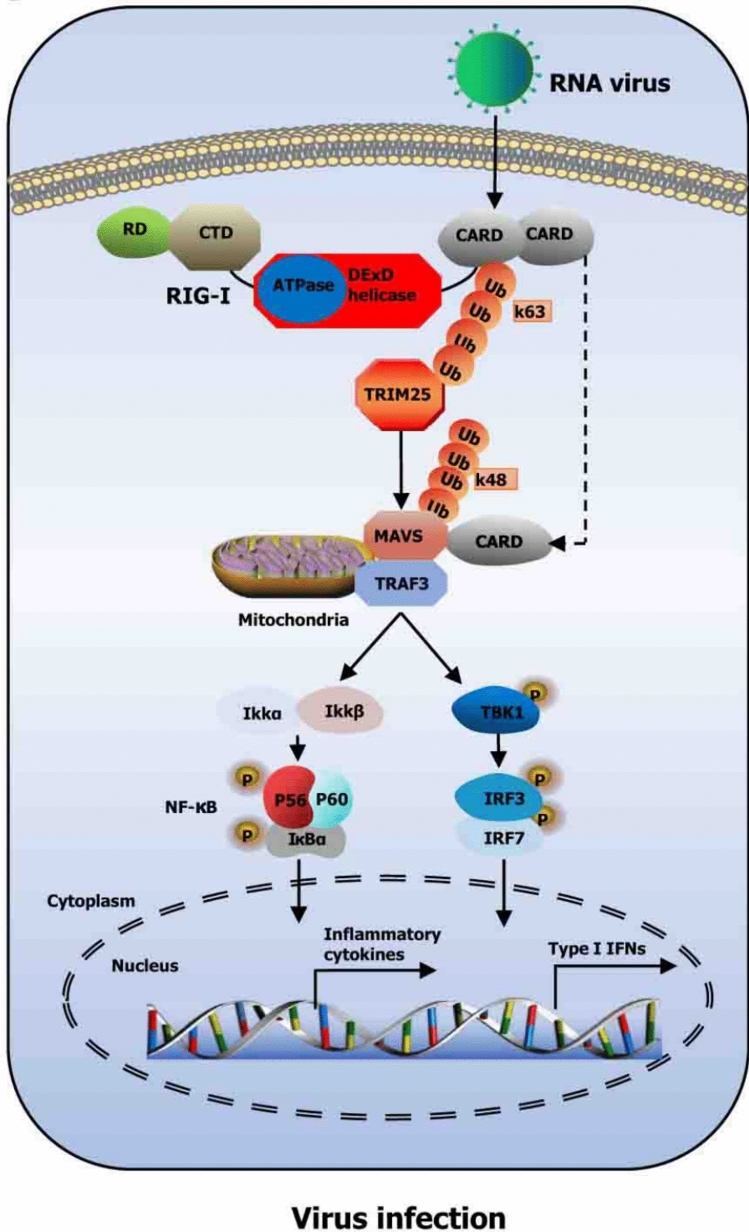
The RIG-I signaling pathway is a critical component of the innate immune response to RNA virus infections. When viral RNA enters the host cell, the RIG-I receptor detects it and undergoes a structural rearrangement that exposes its CARD (caspase activation and recruitment domain) regions. These domains interact directly with the adaptor protein MAVS (mitochondrial antiviral signaling protein), which is anchored to the outer mitochondrial membrane. This interaction triggers downstream signaling involving the activation of kinases such as TBK1 and IKK. These kinases subsequently activate transcription factors, including IRF3, IRF7, and NF-κB. The combined activity of these transcription factors leads to the production of type I interferons and pro-inflammatory cytokines, which help establish an antiviral state in infected and neighboring cells. Copyright © 2022 Liu, Ji, Cheng, Chen, Geng, Huang, Zhang, He, and Song [81] under the terms of the Creative Commons Attribution License (CC BY)

In contrast to TLRs, retinoic acid-inducible gene I (RIG-I) is a cytoplasmic receptor that plays a key role in antiviral defense by recognizing viral RNA (Fig. 2). While RIG-I signaling has not been extensively studied in the transplant setting, its role remains highly relevant, given transplant recipients’ increased susceptibility to viral infections such as CMV and EBV due to long-term immunosuppression. A compromised RIG-I pathway could weaken antiviral immunity, potentially leading to viral reactivation or new infections. Although most of the available data on RIG-I come from broader immunology and virology research, its implications in transplantation medicine, especially in managing post-transplant viral complications, warrant further investigation [81, 104, 117, 151].

### NK Cell Cytotoxic Dysfunction of SOT

**Fig. 3:**
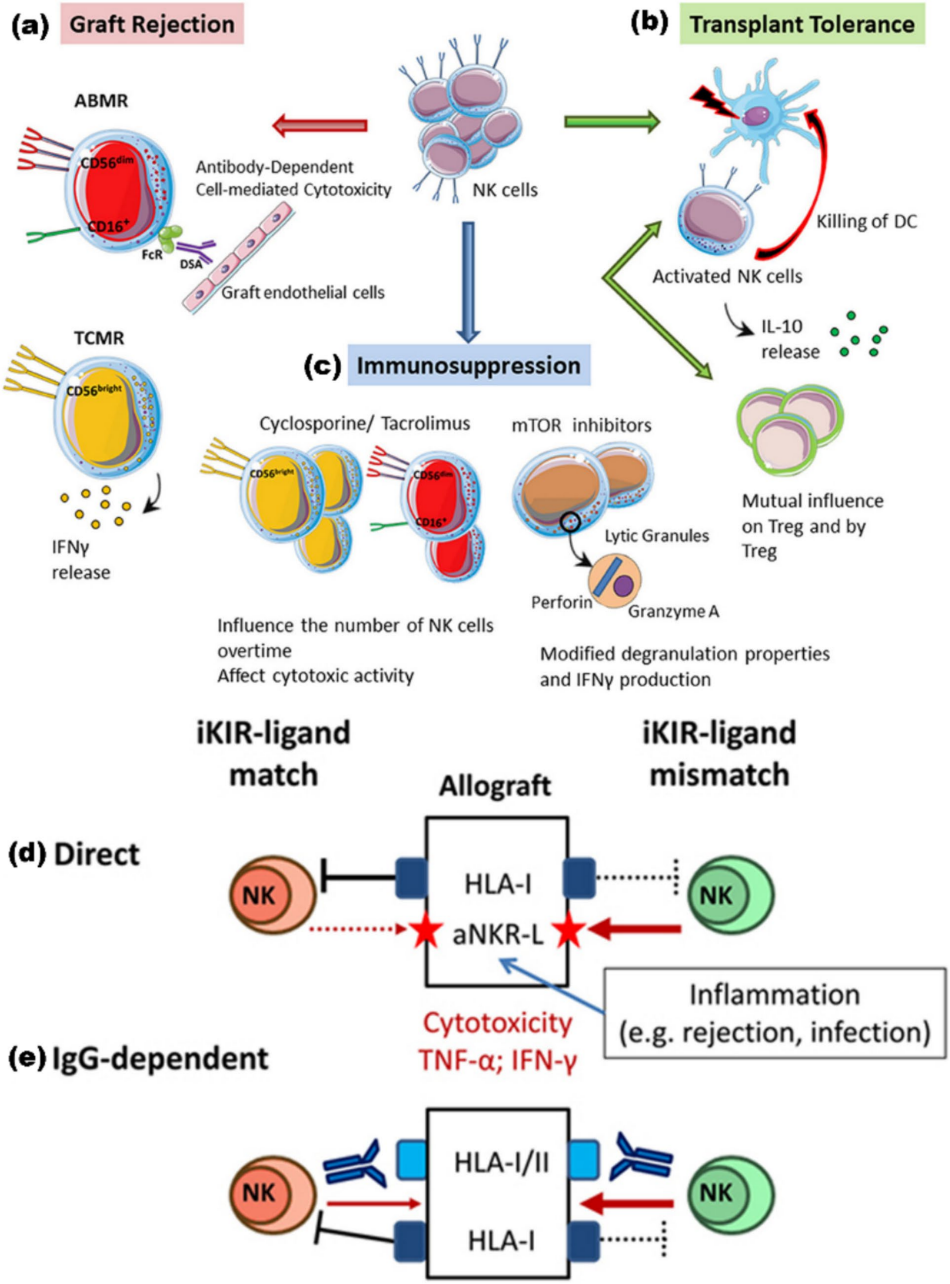
The Multifaceted Role of NK Cells in Transplantation. (**a**) Graft Rejection: NK cells contribute to graft injury through distinct mechanisms, depending on their subsets. CD56dim/CD16 NK cells primarily mediate antibody-dependent cellular cytotoxicity (ADCC). They recognize donor-specific antibodies (DSA) bound to endothelial cells in the transplanted kidney, leading to antibody-mediated rejection (ABMR). In contrast, the CD56^bright^ NK cell subset plays a pro-inflammatory role by secreting cytokines, such as interferon-gamma (IFN-γ), which supports T cell-mediated rejection (TCMR). (**b**) Transplant Tolerance: NK cells can also promote transplant tolerance under certain conditions. One way this occurs is by eliminating donor-derived dendritic cells, thereby reducing antigen presentation and T cell activation [46]. In murine models of transplantation tolerance, NK cells have been shown to secrete interleukin-10 (IL-10), a cytokine with immune-regulatory properties. Moreover, there is evidence that NK cells and regulatory T cells (Tregs) interact in a dynamic, possibly antagonistic relationship that can influence the establishment and maintenance of tolerance. (**c**) Influence of Immunosuppression: Immunosuppressive therapies used after kidney transplantation can affect NK cell function and number. While these drugs may alter NK cell phenotypes, the cells often retain the ability to respond to immune stimuli. However, prolonged immunosuppression tends to reduce overall NK cell numbers, potentially increasing susceptibility to infections or tumor development. Therefore, monitoring NK cell status in transplant recipients can provide insights into immune competence and the risk of post-transplant complications. Copyright © 2020 Pontrelli, Rascio, Castellano, Grandaliano, Gesualdo, and Stallone [113] under the terms of the Creative Commons Attribution License (CC BY). (**d**) NK Cell Alloreactivity and Mismatch: NK cell-mediated recognition of the allograft is also influenced by their interaction with major histocompatibility complex (MHC) molecules. When NK cells lack inhibitory killer cell immunoglobulin-like receptors (iKIRs) for donor-specific HLA class I ligands—a condition referred to as “mismatch”—they may become more prone to launching a cytotoxic response. This effect can be exacerbated under inflammatory conditions that upregulate activating ligands on graft tissues, such as those recognized by NKG2D. (**e**) NK Cell Response to Donor-Specific Antibodies: The presence of DSAs can activate CD16^+ NK cells, prompting them to release cytotoxic molecules and cytokines, even in the presence of inhibitory receptors such as iKIRs. When a mismatch in iKIR-ligand pairing occurs alongside DSA stimulation, the immune response may be further intensified, increasing the risk of graft injury. Copyright: © 2017 López-Botet, Vilches, Redondo-Pachón, Muntasell, Pupuleku, Yélamos, Pascual, and Crespo [84] under the terms of the Creative Commons Attribution License (CC BY)

NK cells are a vital part of the innate immune system, primarily responsible for detecting and eliminating cells that lack self-MHC class I molecules, a mechanism known as “missing self” recognition (Fig. 3). In SOT, NK cells perform a dual function. On the one hand, they contribute to graft rejection by recognizing and attacking donor cells; on the other hand, they serve as a first line of defense against viral infections, particularly in the early post-transplant period when adaptive immunity is suppressed [84, 113]. Experimental studies have highlighted the importance of NK cells in transplant rejection. For instance, in cardiac xenograft models, depleting NK cells using anti-asialo-GM-1 antibodies significantly prolonged graft survival in mouse-to-rat transplants. Clinical observations in humans support this role: patients experiencing acute rejection (grade 3a) were found to have a reduction in circulating NK cells, but an increased infiltration of CD16+ NK cells in kidney graft biopsies, compared to patients with stable grafts (grade 0) [15, 27, 55].

However, the function of NK cells can be significantly altered by immunosuppressive therapies, such as calcineurin inhibitors and corticosteroids. These treatments may blunt NK cell cytotoxicity, thereby compromising their ability to clear viral infections efficiently. Despite this, NK cells exhibit certain memory-like properties. This feature is particularly evident in CMV infection, where a subset of NK cells expressing the Ly49H receptor can enter a dormant state for several weeks and then rapidly expand upon re-exposure to the virus. Such memory-like behavior is essential for long-term immune control but may be impaired in transplant recipients under immunosuppression, increasing their susceptibility to infections [21, 67]. Consequently, in the early (0–1 month) and intermediate (1–6 months) periods, NK-cell cytotoxicity against virus-infected cells is blunted, allowing unchecked CMV replication and EBV-driven B-cell proliferation [106, 109].

### Complement Hijacking

The complement system functions as a frontline defense mechanism in the human immune response. It plays a pivotal role in eliminating pathogens, amplifying inflammation, and marking damaged or foreign cells for destruction. In SOT, however, this well-orchestrated system can inadvertently turn against the graft. Its activation can lead to unintended tissue damage, especially during ischemia-reperfusion injury (IRI), when the restored blood supply to the transplanted organ causes oxidative stress and inflammation. A similar scenario occurs during antibody-mediated rejection (ABMR), in which recipient antibodies target donor antigens on the graft, triggering the classical complement pathway and causing graft injury [116, 122]. Proteins such as C4d and C3d are often detected within the graft tissue within weeks of transplantation. Their presence is not just a marker of ongoing immune activation but also a predictor of future rejection episodes. These complement split products amplify immune responses by enhancing T-cell activation and inflammation, further compromising graft survival [30, 134].

**Fig. 4:**
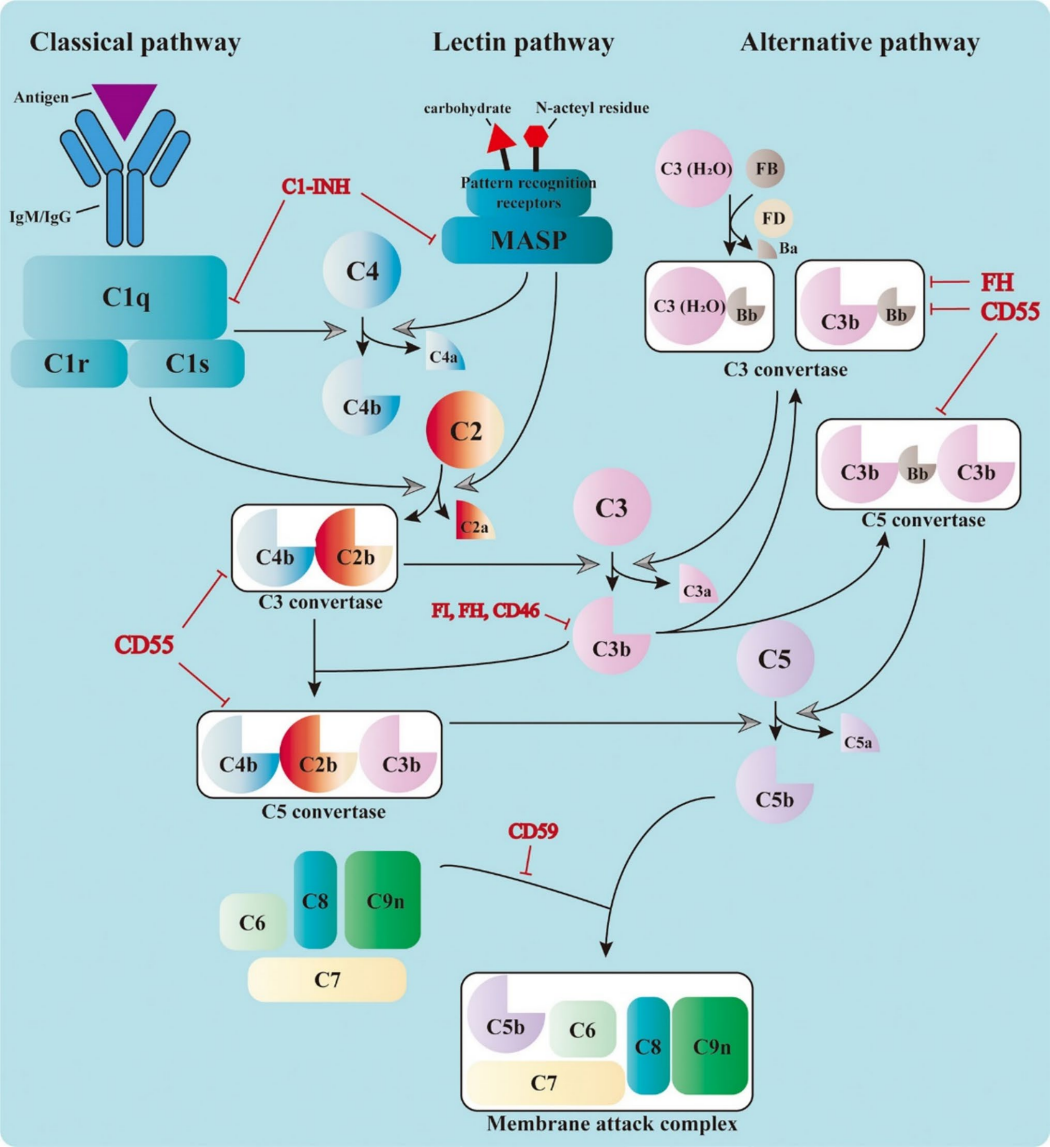
Overview of Complement Cascade Activation**.** The complement system can be triggered through three distinct pathways: classical, lectin, and alternative. The classical pathway is typically activated when C1q binds to antibody-antigen complexes, forming the C1 complex, which then splits the proteins C4 and C2 to create a key enzyme called C3 convertase (C4b2b). Similarly, the lectin pathway is initiated when certain sugar structures on pathogens are recognized by mannose-binding lectin and its associated serine proteases (MASPs), which also cleave C4 and C2 to generate the same C3 convertase. In contrast, the alternative pathway starts with spontaneous activation of C3, forming a different C3 convertase (C3bBb), which is amplified during ongoing immune responses. All three pathways converge at the cleavage of C3, a central event that leads to the production of C3b. This fragment not only helps opsonize pathogens but also contributes to the formation of another enzyme complex, C5 convertase. C5 is then split into C5a and C5b, initiating the assembly of the membrane attack complex (MAC) with the help of additional proteins, such as C6, C7, C8, and C9. This final structure forms pores in the membranes of target cells, ultimately leading to their destruction. Importantly, the complement cascade is tightly regulated by several membrane-bound and circulating proteins to prevent excessive or unintended damage to the body’s tissues**.** Copyright © 2022 Qi and Qin [116] under the terms of the Creative Commons Attribution License (CC BY)

Understanding how the complement system is activated offers key insights into its role in transplantation. It can be initiated via three distinct but converging pathways: the classical, lectin, and alternative pathways. The classical pathway is triggered when C1q binds to immune complexes, leading to the formation of C3 convertase (C4b2b). The lectin pathway is activated when mannose-binding lectin (MBL) recognizes pathogen-associated carbohydrate residues, and downstream MASP proteases facilitate C3 convertase formation. Meanwhile, the alternative pathway is continuously active at low levels and serves as an amplification loop for the other pathways. All three converge on the cleavage of C3 into C3b, which then leads to the formation of C5 convertase and ultimately the membrane attack complex (MAC) that lyses target cells (Fig. 4) [4, 103, 116].

**Fig. 5:**
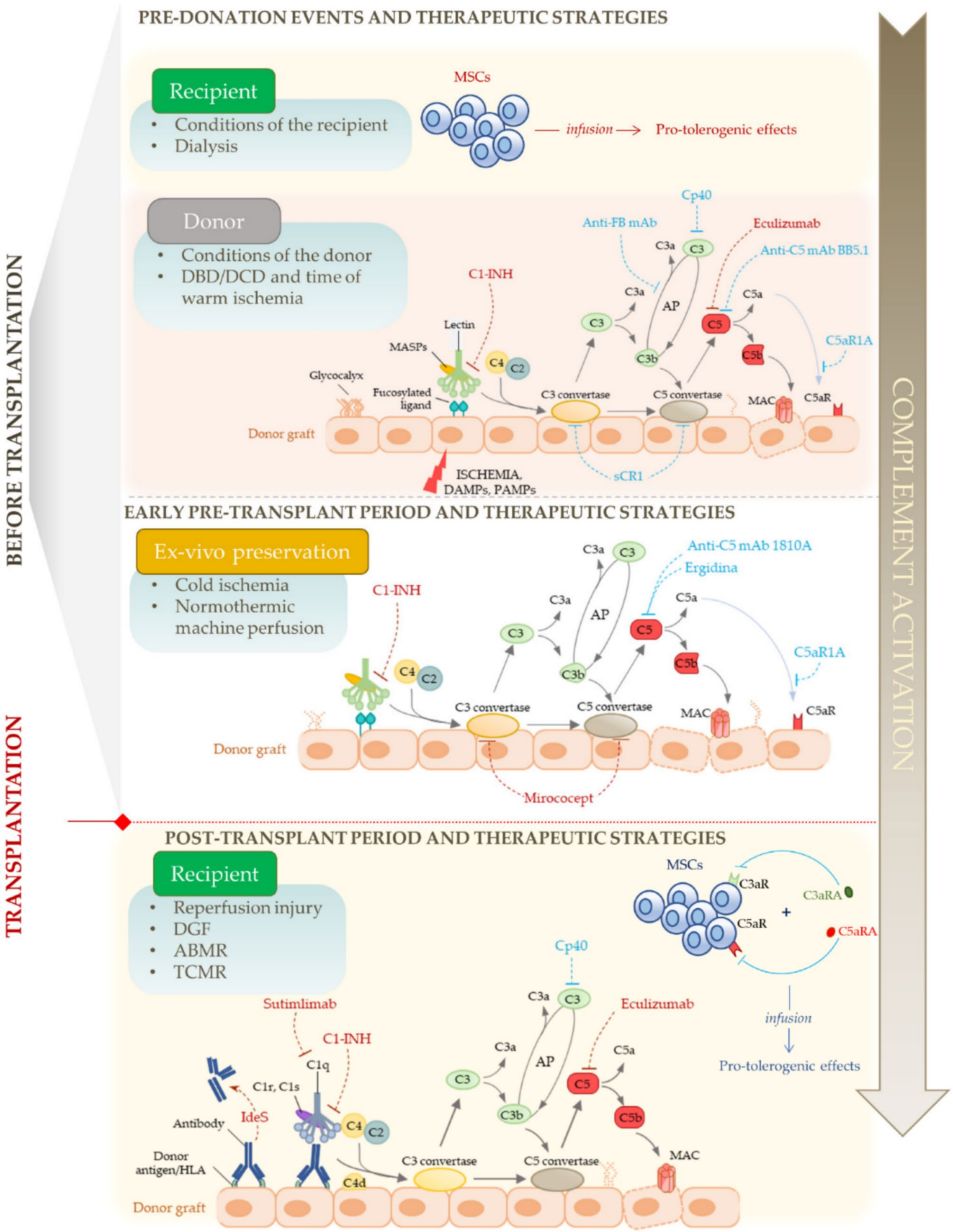
Complement Cascade Activation Throughout Key Stages of Kidney Transplantation. How the complement system becomes involved at various stages of kidney transplantation. Activation may begin even before organ retrieval — both in donors and recipients. For example, underlying health conditions or dialysis treatments in recipients, and systemic issues in donors, can influence the baseline level of complement activation and, ultimately, graft quality. During organ procurement, warm ischemia (particularly in donation after brain or circulatory death, DBD/DCD) and cold ischemia during organ storage trigger the release of danger-associated molecular patterns (DAMPs). These signals, along with increased expression of fucosylated molecules in injured tissue, activate the lectin pathway. This response is further amplified by the alternative pathway. Ischemic injury also compromises the endothelial glycocalyx, leading to a loss of complement regulatory proteins and increased vulnerability to complement attack. Following transplantation, the graft undergoes reperfusion injury and may develop delayed graft function (DGF) and immune-mediated damage. The complement system plays a significant role, particularly in antibody-mediated rejection (ABMR). Here, the recipient’s antibodies bind to donor HLA antigens on the kidney’s surface, initiating classical pathway activation. Although not shown in the diagram, the complement system is also involved in T cell–mediated rejection (TCMR). Emerging strategies, such as the infusion of autologous mesenchymal stromal cells (MSCs) — alone or in combination with inhibitors targeting C3aR and C5aR — have shown promise in reducing complement-driven injury and modulating the immune response. Blue and red dashed lines indicate complement inhibitors that have been explored in preclinical studies and clinical trials, respectively. Reprinted from [122] under the terms and conditions of the Creative Commons Attribution (CC BY) license

During organ donation and procurement, complement activation may already be initiated by pre-existing conditions in the donor or recipient, such as dialysis or systemic inflammation. Ischemic conditions, such as warm ischemia during donation and cold ischemia during storage, further activate the lectin and alternative pathways by releasing danger-associated molecular patterns (DAMPs) and exposing fucosylated molecules. The injury to the endothelium also strips protective glycocalyx components, reducing the graft’s ability to regulate complement activity. Following transplantation, this heightened complement activity contributes not only to early graft dysfunction but also to chronic rejection over time (Fig. 5) [99, 121, 122]. In the setting of immunosuppression, this creates a window for CMV and EBV to exploit complement regulatory mimicry, particularly during the intermediate phase (1–6 months), thereby evading lysis, promoting persistent viremia, and increasing the risk of PTLD [130].

In the setting of immunosuppression, viruses such as CMV and EBV exploit complement regulatory mimicry through a phenomenon termed “complement hijacking.” These viruses produce proteins that mimic or manipulate complement regulatory proteins to evade immune detection and destruction [85, 91]. CMV, for instance, interferes with complement activation, thereby preventing lysis and allowing it to persist in the host [51]. EBV similarly contributes to the risk of PTLD through complement-related immune evasion mechanisms [25, 39, 46]. Encouragingly, new therapeutic approaches are being explored to mitigate complement-mediated injury. For example, the infusion of autologous mesenchymal stromal cells (MSCs), particularly when combined with C3aR and C5aR inhibitors, is being investigated for its potential to suppress anti-donor immune responses and preserve graft function (Fig. 5) [122].

## Adaptive Immunity Compromise

This review focuses on three key ways adaptive immunity is affected in transplant recipients: exhaustion of CD4⁺ and CD8⁺ T cells (linked to increased PD-1 and CTLA-4 expression), impaired B-cell development leading to low antibody levels, and an imbalance between Tregs and Th17 cells [80, 135].

### CD4⁺/CD8⁺ T-Cell Exhaustion (PD-1/CTLA-4 Upregulation)

**Fig. 6:**
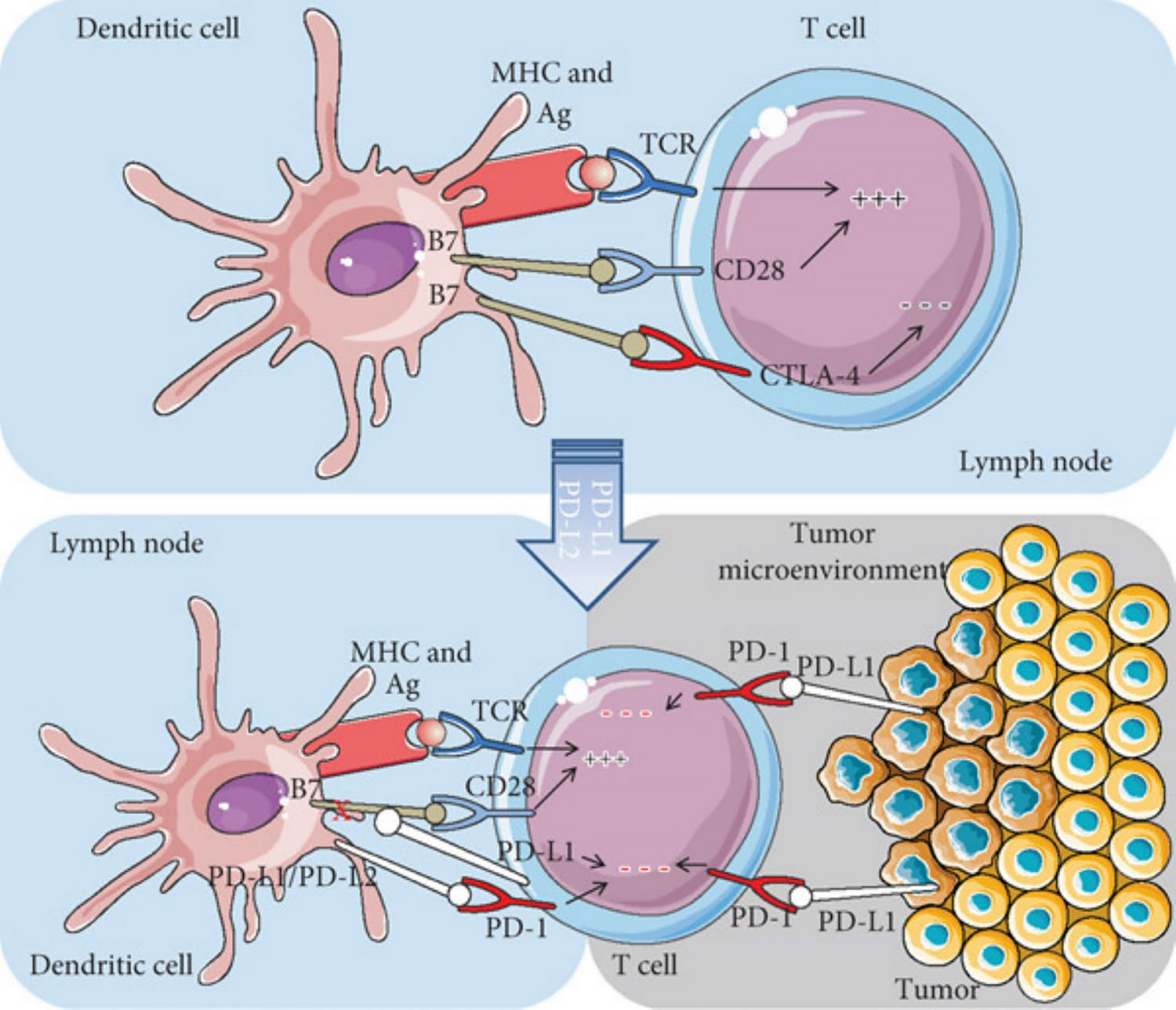
CTLA-4 and PD-1/PD-L1 pathways and the two most important checkpoint inhibitors. Copyright © 2018 Cristina Vajaitu et al. [139] under the Creative Commons Attribution License

T-cell exhaustion is a condition in which T cells, both helper (CD4⁺) and cytotoxic (CD8⁺), gradually lose their functional capacity. These exhausted T cells struggle to multiply, produce signaling molecules like cytokines, and eliminate infected or abnormal cells. This decline often results from continuous exposure to antigens, such as those from a transplanted organ or persistent viral infections like CMV [50, 152]. A hallmark of exhaustion is the increased presence of inhibitory receptors, including PD-1 and CTLA-4, which suppress T-cell activity and are seen in transplant recipients (Fig. 6) [139]. This exhaustion is most pronounced in the late post-transplant period (>6 months). It begins in the intermediate phase, leading to progressive loss of CMV-specific CD8⁺ T-cell effector functions and sustained viremia [145].

One key issue is T-cell exhaustion. Over time, the CD8⁺ T cells that normally control CMV progressively lose effector function, leading to persistent viral replication and an increased risk of complications, such as CMV pneumonitis, particularly in lung transplant recipients [76]. In parallel, B-cell function is frequently impaired after transplantation, leading to hypogammaglobulinemia, characterized by low antibody levels, that compromises control of EBV and markedly elevates the risk of PTLD, a serious complication seen in up to 13% of heart transplant patients [9].

Efforts to reverse T-cell exhaustion, such as immune checkpoint inhibitors (e.g., anti-PD-1 therapies), have proven effective in cancer treatment. However, in transplant recipients, these agents risk reactivating alloreactive T cells, thereby triggering rejection episodes, as documented in clinical reports, especially among kidney transplant patients [17, 62]. Conversely, certain immunosuppressive strategies, such as mTOR inhibitors (e.g., rapamycin), can intentionally promote T-cell exhaustion to enhance graft tolerance [5].

### B-Cell Maturation Defects and Hypogammaglobulinemia

B cells play a central role in the immune system by producing antibodies that neutralize viruses, bacteria, and other pathogens, thereby providing long-term protection. However, in SOT, their function is often disrupted by immunosuppressive medications, such as rituximab (an anti-CD20 antibody) and calcineurin inhibitors [119]. These therapies are crucial for preventing rejection but can impair B-cell maturation and antibody production, leading to hypogammaglobulinemia, a condition characterized by low immunoglobulin levels, especially IgG. This deficiency arises from impaired B-cell development in the bone marrow and secondary lymphoid organs, as well as from disruption of key signaling pathways required for differentiation into antibody-secreting plasma cells [43, 58].

The consequences of B-cell dysfunction are significant. Hypogammaglobulinemia affects around 20% to 40% of SOT recipients, especially within the first year after transplantation. This immune weakness makes patients more vulnerable to infections, including those caused by viruses like CMV and EBV. For instance, when IgG levels drop, the immune system’s ability to keep CMV in check is diminished, increasing the likelihood of CMV-related complications, such as pneumonia or hepatitis, depending on the organ transplanted [108]. B-cell suppression effectively prevents antibody-mediated rejection (AMR) by limiting donor-specific antibodies (DSAs) that activate the complement system; however, it also impairs humoral immunity, resulting in hypogammaglobulinemia and increased susceptibility to infections such as CMV and EBV [20, 128]. The resulting hypogammaglobulinemia, most evident between 1–12 months post-transplant, impairs neutralizing antibody responses against EBV and CMV, directly elevating PTLD and tissue-invasive disease risk [11, 44, 79].

To manage this, clinicians often monitor immunoglobulin levels in transplant recipients, particularly in the early post-transplant period. If low IgG is detected, intravenous immunoglobulin (IVIG) replacement therapy can be administered to reduce infection risk. Clinical studies have shown that IVIG therapy may cut infection rates by up to half in high-risk patients [14, 73, 92, 125]. However, care must be taken not to suppress B-cell function excessively. Newer therapeutic strategies are exploring more selective modulation of B-cell activity, such as targeting the B-cell activating factor (BAFF), to minimize AMR while preserving enough immune competence to fight infections [8, 29].

### Treg/Th17 Imbalance

Tregs, characterized by FoxP3 expression, suppress effector immune responses and actively promote graft tolerance, whereas Th17 cells, which produce IL-17 and express the transcription factor RORγt, drive pro-inflammatory responses that contribute to transplant rejection (Figure 1) [70, 100]. In SOT, the Treg/Th17 balance is frequently disrupted by immunosuppressive medications, ischemia-reperfusion injury (IRI), and viral infections, often shifting toward Th17 dominance and increasing the risk of acute and chronic graft rejection [2].

This equilibrium is tightly controlled by specific transcription factors, FOXP3 and STAT5, which favor Treg differentiation and function. RORγt and STAT3 promote Th17 development, and are further modulated by inflammatory cytokines such as IL-6 and TGF-β, which can convert Tregs into Th17 cells under certain conditions, thereby exacerbating the immune response against the graft [28]. Clinical studies in pediatric liver transplant recipients have shown that elevated Th17 levels correlate strongly with acute rejection episodes, whereas strategies that enhance Treg activity are associated with improved graft outcomes [2]. Agents such as rapamycin, which preferentially support Treg survival over Th17 expansion, have demonstrated these benefits in experimental transplant models [2].

Excessive Th17-mediated inflammation not only heightens the risk of rejection but also impairs antiviral defenses, facilitating viral dissemination and reducing Treg-mediated control of latent viruses such as CMV and EBV [82]. To restore balance, low-dose IL-2 therapy, which selectively expands Treg populations, and anti-IL-17 biologics are under active investigation. In clinical settings involving EBV/CMV viremia, low-dose IL-2 has been shown to rebalance the Treg/Th17 ratio, reduce the risk of rejection, and improve antiviral control without substantially increasing infection susceptibility [150]. Regular monitoring of circulating Treg and Th17 frequencies by flow cytometry, therefore, offers a practical, personalized tool to optimize immunosuppression, simultaneously protecting the graft and limiting opportunistic viral complications [2].

## Viral Immune Evasion Tactics

### MHC-I downregulation (CMV US2-11)

CMV is one of the most common and challenging viral infections in SOT recipients. Infection rates vary widely, from 16% to 56%, depending mainly on the organ type and whether the donor and recipient had prior CMV exposure. This virus poses a serious threat because of its sophisticated ability to evade the immune system, making it difficult to control, especially in immunocompromised individuals [83, 86, 127].

One of CMV’s primary tactics for avoiding immune detection is the suppression of major histocompatibility complex class I (MHC-I) molecules, which generally present viral fragments to CD8⁺ cytotoxic T cells, the immune system’s frontline soldiers against infected cells. CMV uses a group of proteins, specifically US2, US3, US6, and US11, to interfere with different steps of the MHC-I pathway [6]. For instance, US2 and US11 target and degrade MHC-I heavy chains by rerouting them to the proteasome via the ER-associated degradation pathway. US3 disrupts the function of tapasin, a chaperone necessary for proper loading of viral peptides onto MHC-I molecules. Meanwhile, US6 halts peptide delivery by blocking TAP, the transporter that delivers antigenic peptides into the endoplasmic reticulum [53].

This multi-pronged strategy effectively reduces MHC-I expression on the surface of infected cells, making it difficult for CD8⁺ T cells to recognize and eliminate CMV-infected cells. The impact of this evasion mechanism is particularly severe in CMV-naïve recipients (R−) of organs from CMV-seropositive donors (D+). These patients lack pre-existing CMV-specific memory CD8⁺ T cells, so the primary antiviral T-cell response must be generated de novo under heavy immunosuppression; MHC-I downregulation therefore severely impairs antigen presentation, allowing unchecked viral replication and dissemination [86, 127]. Without preventive treatment, infection rates in these high-risk D+/R− patients can reach up to 50%. CMV infection in SOT patients can lead to a wide range of complications. Direct effects include CMV syndrome, characterized by fever, fatigue, and low white blood cell counts, and organ-specific diseases such as pneumonitis, enteritis, encephalitis, and retinitis [68]. Indirectly, CMV can trigger inflammation that intensifies immune responses against the transplanted organ, potentially leading to chronic rejection [12, 52]. For example, in heart transplant recipients, this may contribute to the development of cardiac allograft vasculopathy, a leading cause of long-term graft failure.

To reduce the risk of CMV disease, most transplant centers use antiviral medications like valganciclovir either as preventive treatment or as a preemptive therapy guided by regular monitoring of CMV viral load using PCR. However, CMV’s ability to suppress MHC-I impairs host immune-mediated clearance (but does not alter the mechanism of action of antiviral drugs), often necessitating prolonged courses of antiviral therapy. This therapy, in turn, increases the likelihood of developing drug resistance, particularly against ganciclovir, which can occur in up to 10% of high-risk patients [149].

To overcome these challenges, researchers are exploring new treatment strategies. One promising approach involves the adoptive transfer of CMV-specific virus-specific T cells (VSTs), which are typically polyclonal products containing both CD8⁺ and CD4⁺ populations. While CD8⁺ T cells are MHC class I-restricted, the CD4⁺ component can mediate antiviral effects through MHC class II-restricted cytotoxicity and helper functions, enabling effective viral control even when MHC-I is downregulated [61, 78]. Early clinical studies have shown encouraging results, with reduced viral loads in treated patients. Future management strategies may include routine monitoring of CMV-specific immune responses and the use of newer antiviral drugs, such as maribavir, which have different mechanisms of action and may help bypass existing resistance pathways [87, 90].

### MicroRNA-mediated immunosuppression (EBV BART)

One of the mechanisms EBV uses to evade the immune system and maintain latency involves the production of viral microRNAs (miRNAs), especially those derived from the BamHI-A rightward transcript (BART) region. These small, non-coding RNAs play a critical role in dampening the host’s immune response and creating a microenvironment that allows the virus to persist undetected [22].

BART miRNAs have been shown to interfere with various host immune pathways. For instance, EBV-miR-BART2-5p suppresses MICB expression, a molecule that typically signals natural killer (NK) cells to target infected cells. By reducing MICB levels, EBV effectively evades NK cell-mediated immune surveillance [32, 60]. Other BART miRNAs can inhibit the production of key antiviral cytokines, such as interleukin-12 (IL-12) and interferon-gamma (IFN-γ), weakening the body’s ability to fight the virus [13, 144]. These miRNAs not only function within infected B cells but are also packaged into extracellular vesicles, allowing them to influence immune responses in distant cells, broadening EBV’s immunosuppressive impact [33, 132].

Managing EBV in transplant patients remains challenging. Standard approaches include reducing immunosuppression intensity, administering antivirals such as ganciclovir (which has limited efficacy against EBV), and using rituximab to target B cells in PTLD [1, 142]. However, the presence of EBV-derived miRNAs complicates these interventions by enhancing viral persistence and dampening immune reconstitution. Therefore, more refined monitoring strategies are being investigated, including measuring not only EBV viral loads but also circulating BART miRNAs to identify high-risk patients before complications arise [74, 120]. Emerging therapies aim to target the virus’s immune evasion tactics directly. For example, experimental treatments targeting miRNA inhibitors or EBV-specific T-cell therapies are showing promise in preclinical models by limiting PTLD development and restoring immune function [143], [89]. Understanding of viral miRNA-mediated immune modulation deepens; these novel strategies could pave the way for more effective and personalized care in transplant recipients [33, 132].

## Implications for Controlling Viral Infections in Transplant Settings

Controlling viral infections in transplant settings is vital to mitigate complications and enhance patient well-being. Emphasizing prevention is paramount in managing viral infections among solid organ transplant recipients. Key strategies encompass pre-transplant screening, prophylactic antiviral therapy, and post-transplant viral surveillance to mitigate the impact of these infections. Should prevention efforts falter, early detection coupled with aggressive intervention becomes imperative [45]. CMV stands as a prevalent opportunistic pathogen in transplant recipients, demanding tailored preventive measures contingent upon the serostatus of both donor and recipient. These strategies range from universal prophylaxis to preemptive therapy guided by monitoring CMV DNAemia levels [19]. Recent advances in laboratory monitoring and antiviral therapy have significantly improved outcomes for transplant recipients with viral infections. Swift molecular diagnostic testing, genomics-driven methodologies, and novel antiviral agents are revolutionizing the management of viral infections in transplant settings [96].

## Conclusion

Viral infections remain a significant threat to SOT recipients due to their immunocompromised state, which results from the long-term use of immunosuppressive therapies to prevent allograft rejection. While these agents reduce graft-directed immune responses, residual immune activity, particularly from memory T cells, B cells, and preformed antibodies, can still contribute to graft injury. In this setting, viral infections not only exploit weakened host defenses but can also act as immune triggers, activating innate immune receptors such as TREM-1, TLRs, NLRs, RLRs, CLRs, and the APOBEC protein family, thereby increasing the risk of allograft dysfunction or rejection. Furthermore, viruses like CMV, EBV, and BKV can persist and evade immune surveillance through sophisticated mechanisms, including downregulation of MHC molecules, production of immunosuppressive miRNAs, and interference with antigen presentation. As a result, transplant recipients face not only acute infections but also chronic complications like PTLD or progressive allograft deterioration. To address these challenges, a multifaceted approach is essential. This approach includes rigorous pre-transplant screening, the use of prophylactic or preemptive antiviral therapies, and ongoing post-transplant viral monitoring. Emerging tools such as virus-specific T cell therapies, immune profiling, and complement-targeted interventions are showing promise in enhancing antiviral protection without compromising graft survival. Looking forward, the integration of AI and machine learning holds great potential in predicting viral infection risk, tailoring immunosuppressive regimens, and optimizing individualized patient management. Combining these technologies with rapid molecular diagnostics and real-time immune monitoring will be key to improving clinical outcomes. Future research should prioritize such personalized and adaptive strategies to reduce infection-related morbidity and mortality in transplant recipients, ultimately improving graft longevity and patient quality of life.
